# The characteristics of bipolar dipression

**DOI:** 10.1192/j.eurpsy.2022.1421

**Published:** 2022-09-01

**Authors:** T. Jupe, B. Zenelaj

**Affiliations:** 1 Psychiatric Hospital of Attica, 5th Acute Psychiatric Department, Chaidari, Greece; 2 National Center for Children Treatment and Rehabilitationn, Child Psychiatry, Tirana, Albania

**Keywords:** characteristics, bipolar dipression

## Abstract

**Introduction:**

The National Institute of Mental Health describe the main symptoms of bipolar disorder as alternating episodes of high and low mood. Changes in energy levels, sleep patterns, ability to focus, and other features can dramatically impact a person’s behavior, work, relationships, and other aspects of life. Most people experience mood changes at some time, but those related to bipolar disorder are more intense than regular mood changes, and other symptoms can occur. Some people experience psychosis, which can include delusions, hallucinations, and paranoia.

**Objectives:**

Through this research we aimed to identify all the special features of bipolar depression which will help the clinical psychiatrists in easier diagnosis and management of the disorder.

**Methods:**

Literature review (PubMed)

**Results:**

Clinical Characteristics in Favour of Bipolarity in Depression: psychomotor retardation, history of psychotic depression,history of psychotic depression shortly after giving childbirth, frequent catatonic symptoms, atypical depressive features, severe impairment in interpersonal relationships, inconsistency in business life, history of hypomania, mania or mixed episode,common feeling of numbness and anhedonia; less common sadness and feelings of guilt, mood instability, volatility in temperament, frequent change in affect, daydreaming during the episode and daily life, short duration of depression <3 months, poor cognitive functions during depressive episode, generally similar symptom severity during the day and night etc.

**Conclusions:**

This leads to misdiagnosis of bipolar depression as unipolar depression, which in turn leads to delayed correct diagnosis and treatment and may severely affect the patient’s entire life.

**Disclosure:**

No significant relationships.

